# Assessing the role of contact tracing in a suspected H7N2 influenza A outbreak in humans in Wales

**DOI:** 10.1186/1471-2334-10-141

**Published:** 2010-05-28

**Authors:** Ken TD Eames, Cerian Webb, Kathrin Thomas, Josie Smith, Roland Salmon, J Mark F Temple

**Affiliations:** 1 London School of Hygiene and Tropical Medicine, Keppel Street, London, WC1E 7HT, UK; 2 University of Cambridge, Department of Veterinary Medicine, Madingley Road, Cambridge, UK; 3 National Public Health Service for Wales (now Public Health Wales), Temple of Peace and Health, Cardiff, CF10 3NW, UK

## Abstract

**Background:**

The detailed analysis of an outbreak database has been undertaken to examine the role of contact tracing in controlling an outbreak of possible avian influenza in humans. The outbreak, initiating from the purchase of infected domestic poultry, occurred in North Wales during May and June 2007. During this outbreak, extensive contact tracing was carried out. Following contact tracing, cases and contacts believed to be at risk of infection were given treatment/prophylaxis.

**Methods:**

We analyse the database of cases and their contacts identified for the purposes of contact tracing in relation to both the contact tracing burden and effectiveness. We investigate the distribution of numbers of contacts identified, and use network structure to explore the speed with which treatment/prophylaxis was made available and to estimate the risk of transmission in different settings.

**Results:**

Fourteen cases of suspected H7N2 influenza A in humans were associated with a confirmed outbreak among poultry in May-June 2007. The contact tracing dataset consisted of 254 individuals (cases and contacts, of both poultry and humans) who were linked through a network of social contacts. Of these, 102 individuals were given treatment or prophylaxis. Considerable differences between individuals' contact patterns were observed. Home and workplace encounters were more likely to result in transmission than encounters in other settings. After an initial delay, while the outbreak proceeded undetected, contact tracing rapidly caught up with the cases and was effective in reducing the time between onset of symptoms and treatment/prophylaxis.

**Conclusions:**

Contact tracing was used to link together the individuals involved in this outbreak in a social network, allowing the identification of the most likely paths of transmission and the risks of different types of interactions to be assessed. The outbreak highlights the substantial time and cost involved in contact tracing, even for an outbreak affecting few individuals. However, when sufficient resources are available, contact tracing enables cases to be identified before they result in further transmission and thus possibly assists in preventing an outbreak of a novel virus.

## Background

On May 23rd, 2007, an outbreak of H7N2 influenza in a poultry flock was reported in North Wales, UK. The outbreak was subsequently found to have affected birds at three premises linked to a market held two weeks earlier in North West England. Several human contacts of infected birds developed flu-like symptoms, and the follow-up of exposed people and their close contacts was undertaken, in line with UK guidance [1]. Four individuals (two in Wales) were shown to be influenza A, H7 positive by PCR of whom two (one in Wales) had H7 indistinguishable from the avian strain circulating concurrently (sequenced as A/Chicken/Wales/2007). Here we examine the outbreak in Wales and the response carried out by the National Public Health Service for Wales (NPHS).

Following notification of the avian outbreak by Animal Health, the UK government agency responsible for livestock health, to the public health service, an outbreak control team was convened to lead the response in accordance with UK and WHO guidelines [1]. The response included taking contact histories from individuals who were thought to be cases, and tracing any contacts identified. At the time, stocks of prophylaxis (oseltamivir) were limited, thus there was a need to ensure that it was only given to those at greatest risk of contracting the virus via a contact with a potentially infectious individual.

In communicable disease control, contact tracing is used to link cases together and to identify individuals who may have been infected, and is recommended in suspected human cases of avian influenza [1]. Contact tracing is particularly useful for identifying cases when it is unlikely that infected individuals will independently present themselves for treatment [2,3]. It was particularly appropriate in the outbreak described here because of the non-specific and usually mild symptoms of infection. Contact tracing is a heavy burden for the public health service, especially, as here, when it is not clear beforehand which types of contact may represent a risk. In the absence of any tools for the rapid confirmation of infection by the novel virus and limited availability of prophylactic treatment, the judgement of health-care personnel is the only option to prioritise the response.

A database of the Welsh outbreak was constructed; this database contained details of all cases, suspected cases, and contacts and included information, where available and where relevant, of symptom onset, treatment date, and type of contact. Data were recorded by the NPHS's health protection teams working in collaboration with clinicians in both primary and secondary care settings. We present an analysis of this database, noting that suspected cases are an important element since they contributed to the contact tracing burden.

## Methods

### Case definitions

The outbreak control team used the following case definitions:

A definite case was defined as an individual with laboratory confirmed diagnosis of influenza. A suspected case was defined as an individual with influenza-like illness (ILI) or conjunctivitis AND close contact with either an infected flock, or a definite case, or a suspected case.

ILI was defined as pyrexia (≥38°C) or history of fever AND respiratory symptoms (cough or shortness of breath) with myalgia.

Unless stated otherwise, we will use the term "case" to refer to both definite cases and suspected cases.

### Contact tracing

Close human to bird contact was defined as being within one metre, in a closed environment, with live or dead affected chickens from an affected farm, OR having visited a holding, which had birds with confirmed or suspected avian influenza, on any of the 8 days prior to onset of clinical symptoms.

Close human to human contact was defined as household contact OR social contact for several hours with a human case from 24 hours before to 8 days after onset of that individual's symptoms.

Unless stated otherwise, we will use the term "contact" to refer to either close human or close bird contact.

Contact tracing was carried out either over the phone or face-to-face out from all definite cases and suspected cases. Contact tracing was also carried out from some other individuals who were thought at the time to be possible cases. In instances where cases were not identified until many days after the onset of their symptoms, difficulties with recall meant that it was not possible to identify all of their encounters; all reasonable efforts were made to ensure that contact histories were as complete as possible.

In order to assist in the assessment of the probability of transmission from a "case" to a contact, contacts were divided into three categories, representing expected decreasing levels of risk: home, work, and other. "Home" describes the location where the patient usually slept; "work" describes the patient's place of work (or education); "other" contacts includes encounters with individuals who were on the same hospital ward or the same doctor's surgery as cases. Not all interactions fitted neatly into these categories; in such instances, the most appropriate option was chosen.

The offer of prophylaxis to contacts was made to those who met the definition of a close contact in the opinion of the health professional examining them. Contacts who were not traced until many days after their exposure and who were then still without symptoms were not given treatment.

### Social networks

The outbreak and the contact tracing efforts can be visualised by plotting the social contact network of the outbreak; such a network plots individuals as points and joins with lines those individuals who have been linked through contact tracing [4,5]. Plotting the outbreak as a network has been used to assist in the identification of individuals likely to be at increased risk of infection, and aids the evaluation of the likely source of infection [6,7].

## Results

Once the public health service had been alerted to the outbreak, on May 23rd, contact tracing was carried out from individuals who were thought to be cases. By the end of May 27^th ^full contact histories had been obtained from 12 individuals. The 142 distinct contacts identified were telephoned and those with symptoms were examined by a specially convened team of local general practitioners, all using Personal Protective Equipment (PPE). Field epidemiology, including obtaining the history of both clinical symptoms and contacts, was carried out by the outbreak control team and collated by the support team from the Communicable Disease Surveillance Centre (Wales).

One week after the initial notification, 20 individuals who were thought to be cases and 236 distinct contacts had been identified. Contact tracing and the administration of antiviral drugs were continued where necessary, and the outbreak was declared over on June 5^th ^2007.

Subsequently, either on the basis of alternative virological diagnoses being confirmed by laboratory tests (one of parainfluenza 3 and one of rhinovirus) or as further information became available indicating that that the individuals concerned had had no exposure to infected birds nor to other cases, the total number of human "cases" in the Welsh outbreak was reduced to 14.

In total, contact tracing was carried out from 86 people and 3 groups of livestock (2 flocks and one market). The 14 "cases" identified 281 contacts (not all distinct) while 72 others, who were subsequently determined not to be cases, identified 337 contacts (not all distinct). In total, 254 distinct individuals (cases and contacts) were identified. 102 individuals were given treatment or prophylaxis; 86 courses of antiviral drugs were provided. The control and contact tracing efforts involved approximately 60 public health personnel.

**Figure 1:**
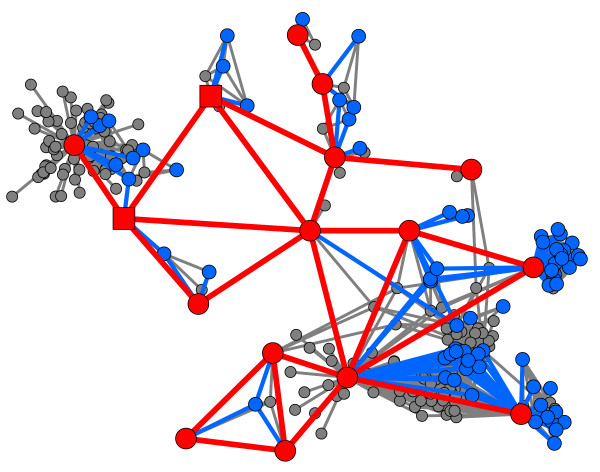
**Outbreak network**. Network showing the individuals and poultry involved in the outbreak and the links identified between them. The network contains 254 individuals, (including 2 cases in poultry (squares), 14 human cases, and 72 other individuals from whom contact tracing was carried out). Cases and links between cases are shown in red. Those other individuals from whom contact tracing was carried out and their links to cases are shown in blue. Other individuals and links are shown in grey.

**Figure 2:**
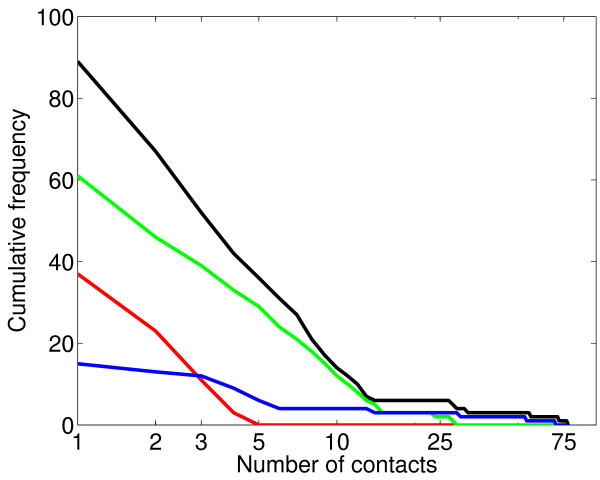
**Population variability in number of contacts identified**. For each point on the x-axis, the black line shows the number of people (out of the 86 individuals from whom contact tracing was carried out) who identified at least that many contacts. The red, green, and blue lines show the same for home, work, and other contacts respectively.

The cases and contacts identified were linked together through a network of social contacts (Fig 1). The network displays the wide variability in the number of contacts identified by this population: while many individuals had low numbers of potentially risky contacts, other individuals were extremely well connected (Table 1, Fig 2), a result observed in many other epidemiologically relevant networks [5,6,8]. As might have been anticipated, the range in number of contacts identified was lowest at home, whilst some individuals identified large numbers of contacts at work and "other" locations.

**Table 1:** Summary data of cases, dates of symptom onset, times to treatment, numbers of contacts, and estimated secondary cases in the absence of treatment.

Case	Date of symptom onset (days after May 1st)	Days between appearance of symptoms and treatment	Number of contacts identified			Estimated secondary cases in the absence of treatment
				
			Home	Work	Other	
1	11	13	3	4	69	1.1

2	17	7	1	2	4	0.3

3	18	6	3	1	4	0.5

4	20	4	4	3	5	0.8

5	21	3	4	14	53	2.1

6	22	3	2	1	0	0.3

7	22	7	3	1	0	0.4

8	23	3	3	23	29	2.7

9	25	0	1	14	13	1.5

10	26	0	1	0	2	0.1

11	26	1*	4	0	3	0.5

12	28	1	2	0	0	0.2

13	28	0	3	0	0	0.3

14	28	0	2	0	0	0.2

*prophylaxis was contraindicated in this individual; symptoms were identified 1 day after appearance and the individual ceased social activities at this point.

**Figure 3:**
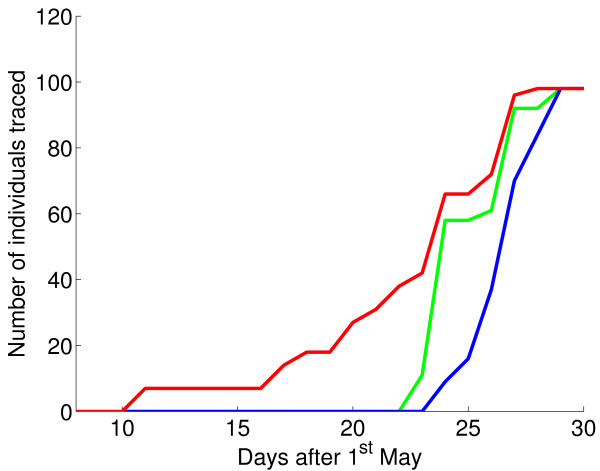
**Actual and idealised time courses of treatment and tracing**. Time course of tracing and treatment/prophylaxis during the outbreak (blue). Also shown are two idealised situations: one - instantaneous tracing (green) - in which tracing and treatment/prophylaxis take place immediately a contact is named; the other (red) in which there is both instantaneous tracing and perfect surveillance, such that as soon as individuals become symptomatic they are treated and their contacts are identified and traced.

Once contact tracing began in earnest, it rapidly caught up with the spread of infection (Table 1, Fig 3). In an ideal scenario, infected individuals would be identified immediately they displayed symptoms (perfect surveillance) and all contacts would be traced immediately they had been identified (instantaneous tracing), but in reality this is never possible. It is hard to quantify the impact of any delays in contact tracing when the inherent properties of the infection (latent period, transmissibility, potential for transmission before onset of symptoms) are unknown. In this case, the public health imperative was to assume the worst case scenario of a previously unencountered human infection. In Fig 3 we see how quickly those traced individuals eventually treated or given prophylaxis could have been reached in the idealised case of perfect surveillance and instantaneous tracing (of course, we would anticipate that under conditions of perfect surveillance and instantaneous tracing the outbreak would have ended sooner and the number of doses of treatment/prophylaxis would have been reduced). We see that the principal hold-up came from the two week delay in recognition of the outbreak in poultry, with its potential risk to humans. Contact tracing, once the potential serious public health implications were realised, was extremely rapid.

**Figure 4:**
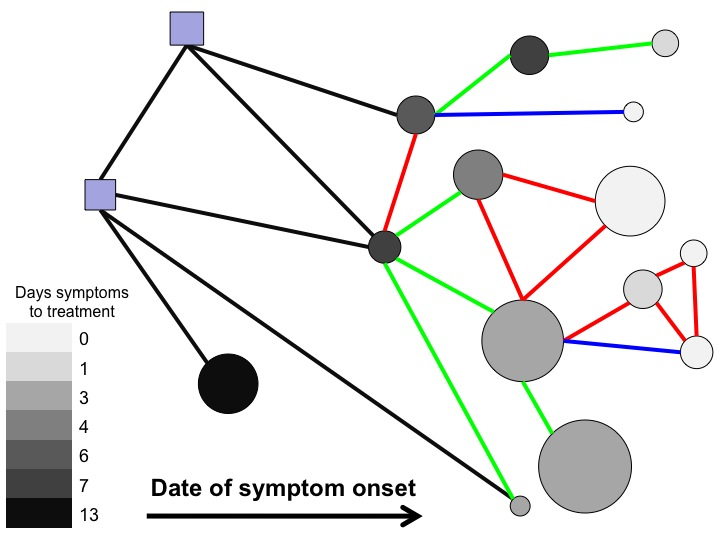
**Time-ordered network of linked cases showing estimated secondary infections in absence of treatment**. The 14 human cases (circles), along with the infected market and flock (squares), plotted from left to right in time ordered position; also shown are traced links between them categorised as home (red), work (green) and other (blue). Links involving birds are in black. The colour of a circle shows the length of time between the onset of symptoms and treatment being given, while the area is proportional to the approximate expected number of subsequent cases had they not been treated.

In this outbreak the response was sufficient for tracing efforts to catch up with suspected infection, so that later cases were treated almost immediately they exhibited symptoms (Table 1, Fig 4). Assuming that person-to-person spread could have occurred, this aided control by reducing both the opportunity for ongoing infection and the number of contacts that needed to be traced.

If all the human cases were indeed caused by the same infective agent, such as H7N2 influenza, the most straightforward explanation of the epidemiological data from case interviews is that 3 infections were acquired directly from infected birds, 4 from household contacts, 6 from workplace contacts and 1 from other contacts. Since the cases identified a total of 36, 63, and 182 home, work, and other contacts respectively, this allows us to estimate the probability of transmission in the three different settings as 0.11, 0.095, and 0.0055. Unsurprisingly, contacts at home appear to be the most risky, with those at work also considerably more risky than those in "other" settings. We can use these estimates and the details of the contacts identified by each case to estimate the number of secondary cases that each case might be expected to generate (Table 1, Fig 4) [9]. As anticipated, there is considerable heterogeneity; the expected number of secondary cases ranges from 0.1 to 2.7. Such an exercise helps to highlight those individuals who, in the event of person-to-person spread, might be expected to have caused further cases. That cases late in the epidemic generated few secondary cases could be explained as the result of the speed with which they were contacted by the outbreak control team. Being treated rapidly after symptoms appeared or receiving prophylaxis before symptoms appear would, in general, in any disease in which person-to-person spread occurred, be expected to reduce the opportunity to infect contacts.

## Discussion

The outbreak occurred in a highly heterogeneous population, both in terms of contact structure and risk for further spread. Home and workplace contacts accounted for most of the suspected cases, with "other" settings being far less risky. The heterogeneities seen are partly due to behavioural differences and partly due to variation in speed of diagnosis. For instance, case 1 was hospitalised but not isolated for almost a fortnight before being diagnosed and therefore acquired an unusually large number of contacts (although none became cases). School pupils or teachers similarly would usually be expected to have had many contacts [10], but fortunately the main outbreak took place over a Bank Holiday weekend and subsequent half term holiday.

Difficulties in obtaining data and mobilising personnel, and the fact that the outbreak in poultry did not immediately come to light, meant that infection initially spread with the public health service having little idea where it was. The problem was exacerbated by the non-specific nature of the symptoms of the infection, which meant that some cases were not identified until they had been traced from other cases. However, once the outbreak was detected, contact tracing allowed the rapid identification of at-risk individuals. This reduced the delay between the onset of symptoms and the application of treatment/prophylaxis; contact tracing could be plausibly identified as having lowered opportunities for further spread.

Resources for contact tracing were locally stretched in the few days following the notification of the outbreak. However, the outbreak control team was able to draft in the extra resources required; contingency plans were in place to allow further support to be enlisted from other parts of Wales, but this was not necessary. There were limited supplies of prophylactic treatment at the time of the outbreak, although this is no longer the case as the worldwide production and stock has increased.

It was not clear that there was a single source of infection, as there was laboratory confirmation of Influenza A in only 2 of the "cases". It is possible that there were several unrelated causes of their symptoms, and hence it is possible that more than one organism accounted for the pattern of illness observed in this social network.

It is not clear whether H7 Influenza A, as observed here, is sufficiently infectious to have spread widely had it been allowed to proceed without any attempt being made at control. Indeed, some doubt whether avian influenza strains including this one can spread from person to person at all. In this outbreak, due to the lack of diagnostic test results, it is not possible to be certain as to the organism responsible. Only two patients had positive PCR tests for H7 and none had diagnostic seroconversion. Nevertheless, the data analysed are consistent with limited chains of person-to-person transmission, a phenomenon observed in the Netherlands during an H7N7 outbreak in 2003 [11,12]. Other contemporary North American influenza H7 viruses possess human receptor specificity, suggesting that H7 strains may be more transmissible than other avian strains [13]. Thus, in any real-time outbreak of a novel infection, it would be unwise to assume, *a priori*, that person to person spread could not occur, especially given the possibility of successive generations of human infection evolving to become more efficiently transmitted [14]; the outbreak described here was therefore treated as a novel human influenza strain derived from an avian source, and therefore illustrates the possible burden of intervention necessary in future similar outbreaks.

## Conclusions

Any avian influenza virus (whether of high or low pathogenicity in birds) could be a plausible trigger for a major human pandemic if it can spread from birds to humans, spread effectively from person to person, and if it becomes highly pathogenic in humans. This was the rationale for the major public health contact tracing response described here. The same precautionary response would be required in similar situations in future. The outbreak thus provides a natural experiment for interventions that have been envisaged in pandemic planning for the early stages of pandemic influenza [1]. Contact tracing is a powerful intervention [3,15] that will always have a role as a means of gathering information about an epidemic but may not be able to control infection. Contact tracing can only be carried out effectively with well-organised personnel, good data management, and sufficient expertise in the field to make rapid decisions.

## Competing interests

The authors declare that they have no competing interests.

## Authors' contributions

KT, JS, RS, and MT compiled the outbreak dataset. KE and CW performed the network analysis. KE wrote the manuscript with input from all authors. All authors read and approved the final manuscript.

## Pre-publication history

The pre-publication history for this paper can be accessed here:

http://www.biomedcentral.com/1471-2334/10/141/prepub
